# Mapping barrier island soil moisture using a radiative transfer model of hyperspectral imagery from an unmanned aerial system

**DOI:** 10.1038/s41598-021-82783-3

**Published:** 2021-02-08

**Authors:** Rehman S. Eon, Charles M. Bachmann

**Affiliations:** Rochester Institute of Technology, Chester F. Carlson Center for Imaging Science, Rochester, NY 14623 USA

**Keywords:** Environmental sciences, Hydrogeology, Infrared spectroscopy, Near-infrared spectroscopy, Spectrophotometry, Imaging techniques

## Abstract

The advent of remote sensing from unmanned aerial systems (UAS) has opened the door to more affordable and effective methods of imaging and mapping of surface geophysical properties with many important applications in areas such as coastal zone management, ecology, agriculture, and defense. We describe a study to validate and improve soil moisture content retrieval and mapping from hyperspectral imagery collected by a UAS system. Our approach uses a recently developed model known as the multilayer radiative transfer model of soil reflectance (MARMIT). MARMIT partitions contributions due to water and the sediment surface into equivalent but separate layers and describes these layers using an equivalent slab model formalism. The model water layer thickness along with the fraction of wet surface become parameters that must be optimized in a calibration step, with extinction due to water absorption being applied in the model based on equivalent water layer thickness, while transmission and reflection coefficients follow the Fresnel formalism. In this work, we evaluate the model in both field settings, using UAS hyperspectral imagery, and laboratory settings, using hyperspectral spectra obtained with a goniometer. Sediment samples obtained from four different field sites representing disparate environmental settings comprised the laboratory analysis while field validation used hyperspectral UAS imagery and coordinated ground truth obtained on a barrier island shore during field campaigns in 2018 and 2019. Analysis of the most significant wavelengths for retrieval indicate a number of different wavelengths in the short-wave infra-red (SWIR) that provide accurate fits to measured soil moisture content in the laboratory with normalized root mean square error (NRMSE)< 0.145, while independent evaluation from sequestered test data from the hyperspectral UAS imagery obtained during the field campaign obtained an average NRMSE = 0.169 and median NRMSE = 0.152 in a bootstrap analysis.

## Introduction

In recent years unmanned aerial system (UAS) technology has increased the accessibility and impact of remote sensing imagery in a wide variety of applications^1^ including environmental science^2^, monitoring and stewardship^3,4^ of coastal zone management^5^, agriculture^6–8^, defense^9^, and industry^10–12^. The ability to rapidly deploy and analyze data from such platforms allows for greater flexibility and the possibility of frequent data acquisitions at low cost that would not otherwise be possible from conventional aircraft. Many types of remote sensing systems have been flown on UAS platforms^13^ including thermal^7,14,15^, multi-spectral^5,14,15^, hyperspectral^15,16^, LiDAR^15–17^, and more recently synthetic aperture RADAR (SAR)^18,19^. While multi-spectral and LiDAR have been among the more frequently used imaging systems, lower-weight imaging spectrometer systems that can be used on UAS platforms are now becoming standard. For geophysical mapping of surface conditions, hyperspectral imagery has a distinct advantage over conventional RGB and multi-spectral imagery due to greater information content. Soil moisture content (SMC) is an important geophysical variable that plays a critical role in many of the applications just listed, and accurate retrieval of this geophysical parameter, therefore, is an important goal. Hyperspectral imaging provides the possibility of more accurately quantifying SMC for these applications, and the use of such imaging systems onboard UAS provides a means of more cost-effectively mapping SMC.

In sediment, retrieval of moisture content is complicated by the fact that water occupies a pore space of variable size within a matrix of potentially variable composition. A number of approaches have been taken to model soil moisture content from spectral data, including the use of spectral indices^20–22^, band depth of water absorption features^20,23^, and simplified radiative transfer models, such as those based on a two-stream approximation for diffuse reflectance using the Kubelka–Monk model^24,25^. In this work, we employ a recently developed radiative transfer model that incorporates the possibility of a directional source and has produced promising results in a laboratory setting^26,27^. While we considered more than one of these alternative approaches that could in principle be applied to hyperspectral imagery, the primary models that address the problem at hand of modeling water in the sediment pore space were the two-stream approach based on Kubelka–Munk (K–M) theory^24^ and the model which we analyze in depth in this work, the multilayer radiative transfer model of soil reflectance (MARMIT)^26,27^. Comparisons of these two approaches demonstrated that MARMIT obtained more robust results. This is not surprising given the fact that Kubelka–Munk theory is a model of diffuse reflectance and does not address the directional nature of the solar source, while MARMIT does incorporate a directional source. Sadeghi et al.^24^ did describe an alternative version of their K–M based approach that could incorporate directional sources through use of the Fresnel equations to describe directional illumination, however, their analysis nonetheless excluded this modification due to difficulty of physical interpretation. As we will describe below, while MARMIT does require a calibration step, Bablet et al.^26^ do suggest potential physical interpretations for some of the calibration parameters, and in addition, here, we present evidence that the model does have some ability to generalize beyond an immediate locale. To that end, in this paper, our bootstrap validation analysis considers two distinct sites separated by $\sim$ 0.25 km from two field campaigns conducted in different years (2018 and 2019). The fact that the bootstrap analysis incorporated both spatially and temporally disjoint data supports the conclusion that MARMIT does allow for generalization beyond immediate circumstances. Given all of these factors, in this work, our analysis focuses on MARMIT.

MARMIT^26,27^ makes the simplifying assumption that the embedded SMC can be treated by means of a layer of equivalent water thickness, with an assumed efficiency parameter, which weights the fraction of wet surface area present in a given location. The model treats soil and water as separate layers and uses the familiar Fresnel formalism for reflection and transmission at the boundaries of both the soil layer and the equivalent water layer. Successive orders of internal reflection and transmission factors modeled by Fresnel coefficients at the sediment/water boundary and air/water boundary appear in the model along with absorption modeled using the Beer–Lambert law along each transit between boundaries. To date MARMIT has been applied only to laboratory reflectance measurements from point spectrometers^26^ and laboratory-based hyperspectral imaging systems^27^. In the present work, we validate this model in a field setting from a UAS platform^15^ and undertake additional laboratory tests across a set of disparate sediment types, varying in composition, surface roughness, and grain size distribution, derived from different locations within the U.S. to demonstrate the promise of this approach for practical use. In its current form, MARMIT is appropriate for bare soil SMC retrieval. In the future, MARMIT in principle could be adapted to work with vegetated surfaces, however to do so, would require the addition of a layer of vegetation within the model.

## Results

### Spectral analysis

**Figure 1:**
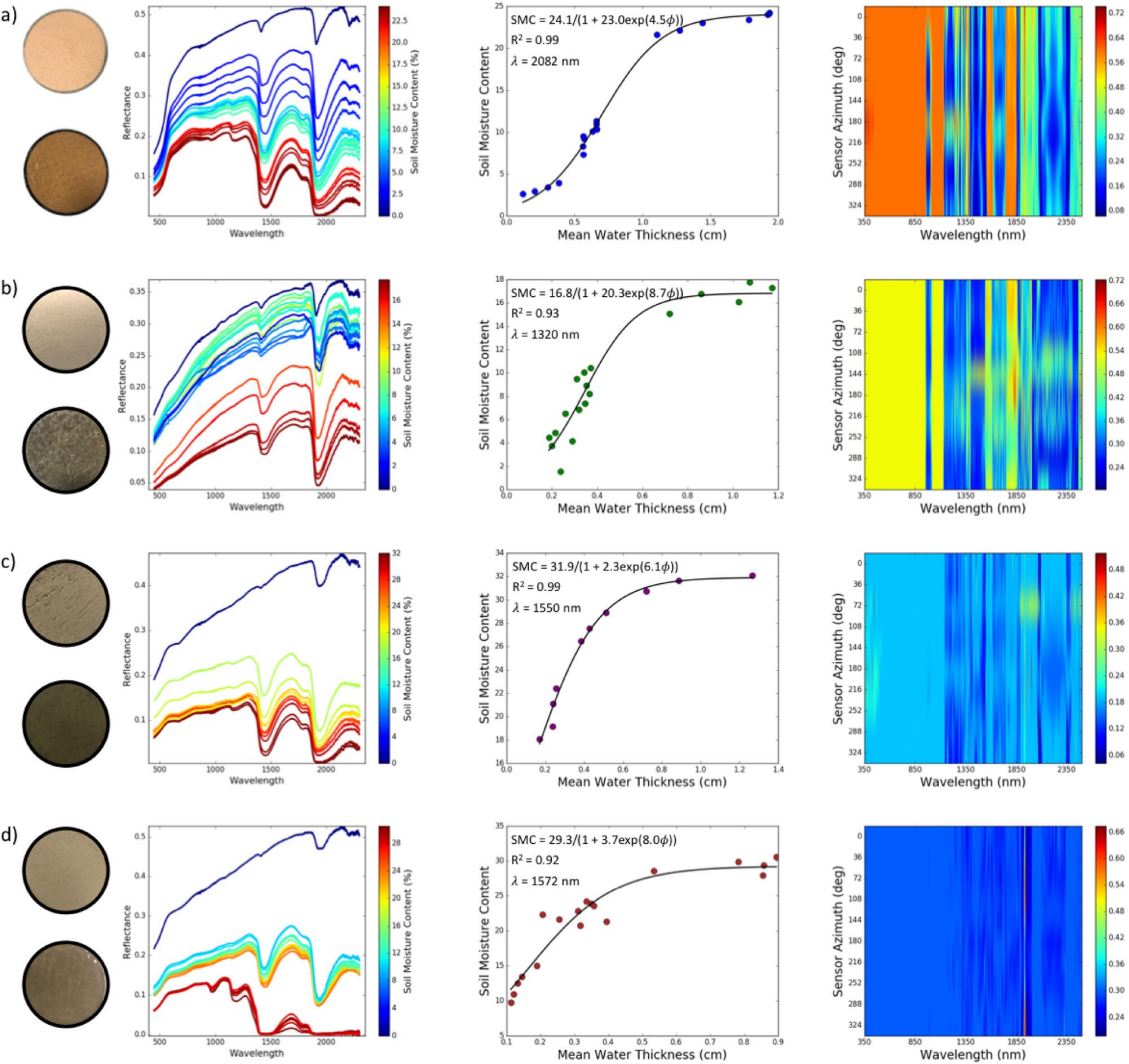
(First column) Photos of dry (top photo) and wet (bottom photo) soil samples for the four sites: (**a**) Algodones, (**b**) Nevada, (**c**) Hog Panne, and (**d**) Hog Beach. (Second column) Corresponding spectral reflectance plots of the four types of sediments at a sensor orientation with zenith angle = $0^\circ$ and relative azimuth angle (to the light source) = $36^\circ$ over a progression of moisture levels; color of reflectance curves corresponds to moisture level (right colorbar of each plot). (Third column) Best model fit found, over all possible combinations of wavelength and sensor zenith/azimuth orientations, relating SMC and mean water thickness (range of NRMSE = 0.044–0.145 and regression coefficient of determination^28^, $R^2$ = 0.92–0.99). (Fourth column) NRMSE of all models as a function of sensor relative azimuth and wavelength with color key representing the error level.

Figure 1 shows the evolution of the soil spectra as a function of SMC for the four distinctly different laboratory soil types. For all soil types, the reflectance is highest when completely dry. In the dry state, the reflectance increases with wavelength from visible to the infrared part of the electromagnetic spectrum. As the SMC increases, we notice a decrease in reflectance across all wavelengths, with the drop in reflectance being more evident in the infrared. The change in moisture content is most pronounced at the two major water absorptions bands at 1440 nm and 1930 nm. We also observe weak water absorption features at 970 nm and 1160 nm for some of the spectra. This is prominent in the high-SMC reflectance spectra for the HOGB sediment sample (Fig. 1d). The change in band depth is more pronounced in the SWIR and clearly nonlinear compared to the visible part of the spectrum. However, from our experiments, we notice that the reflectance does not always decrease with an increase in water content. For example, the NEV soil does not follow this trend for some of the low SMC spectra. The NEV soil sample had high clay content, and physical changes in this sediment with drying are typically different than sediments with lower clay fraction. The spectra for 6–8% SMC are higher than the reflectance at SMC values of 2–4%, which is not consistent with the other soil types. The rate of change in reflectance is different for each of the sediment types as well. We see a big drop in reflectance for the HOGP (Fig. 1c) and HOGB (Fig. 1d) samples. The different rates of change in reflectance as a function of SMC were partially influenced by the time in between each measurement. Of the 4 soil samples, the HOGB sample was the only soil sample in the study that reached complete saturation. This level of saturation was evident with nearly complete absorption or almost zero reflectance in the SWIR at the high SMC values. The complete absorption of the spectra is only evident in the strong absorption features (1440 nm and 1930 nm) for the other three data sets that did not reach saturation. The strong change in absorption in the SWIR bands as a function of SMC suggest that this wavelength region will be most effective for the characterization of water content using the MARMIT model.

### SMC retrieval using MARMIT

**Figure 2:**
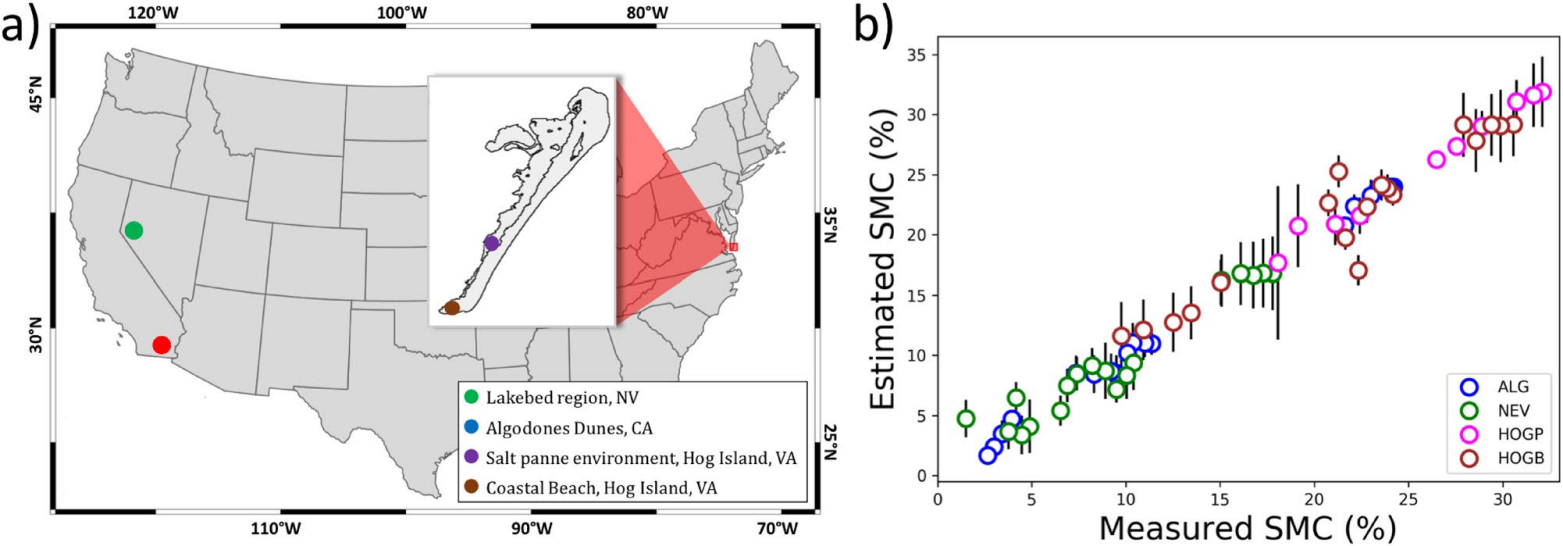
(**b**) Relationship between the estimated model SMC and measured SMC for laboratory samples from the four field test sites: (**a**) Algodones (ALG), Nevada (NEV), Hog Island Panne (HOGP), and Hog Island Beach (HOGB). (**b**) Circles represent the best matched moisture obtained across all sensor zenith and azimuth measurements and wavelength combinations, while y-error bars represent the variation of the SMC output by the model at a specific wavelength over the spectral reflectance recorded by GRIT-T over all zenith and azimuth angles.

SMC retrieval in MARMIT depends on fitting a logistic function relationship between the mean equivalent water thickness and SMC (Eq. 5). Bablet et al.^26^ explain that it is not possible to determine a unique relationship between equivalent water thickness and SMC that is valid for all types of sediment. The variation in the sediment characteristics, as well the methodology used in preparing the samples for the laboratory analysis (e.g. the sediment wetting and drying protocol), has a significant influence on the relationship between mean equivalent water thickness and SMC. As a result, we developed a model for each sediment sample separately, with a unique logistic function relating the SMC to the equivalent water thickness for each sediment type. Figure 1(column 3) shows the S-shaped logistic curve for each of the four different soil types. The logistic functions shown represent the model for the wavelength where we obtained the best fit for each of the different soils. The best fit obtained depended on the sample: 2082 nm, 1320 nm, 1550 nm, and 1572 nm for the ALG, NEV, HOGP and HOGB soil samples respectively. For these wavelengths, the accuracy of the fit was very high with normalized root mean square error (NRMSE) in the range $NRMSE < 0.145$; in this work, we define NRMSE to be the ratio of mean squared error to mean measured value^29,30^, also sometimes referred to as the “Scatter Index”^29,31^, in a given bootstrap trial: $NRMSE = \frac{ \sqrt{\frac{1}{N}\sum _{i=1}^N (SMC_{i,pred} - SMC_{i,gt})^2 } }{{\overline{SMC}}}$, where $SMC_{i,pred}$ and $SMC_{i,gt}$ represent respectively the model prediction and measured (ground truth) values of the SMC for the *ith* sample in the specific bootstrap test and ${\overline{SMC}}$ represents the average ground truth SMC value over the same set of bootstrap samples. While these were the best wavelength models found in each case, there were several wavelengths where the NRMSE was relatively low in the SWIR range, so that good model fits could be obtained for more than one wavelength. In contrast, the accuracy of the fit between SMC and mean equivalent water thickness was very poor in the VIS-NIR wavelength range. The fourth column of Fig. 1 illustrates these points by comparing the NRMSE for each sediment sample for the complete wavelength range (350–2500 nm) and the sensor azimuth of the hyperspectral goniometer system, the Goniometer of the Rochester Institute of Technology-Two (GRIT-T)^32^. We only observed a small variation in the NRMSE across view-geometry. Interestingly, the NRMSE varied more with sensor azimuth compared to sensor zenith. The NRMSE plots show that the accuracy in fitting SMC to mean equivalent water thickness using the MARMIT model depended more on the wavelength than on the observation direction of the reflectance and that a number of different wavelengths in the SWIR would be suitable for accurately modeling the data. A plot of the measured SMC versus the estimated SMC for all sediment samples from the laboratory study appears in Fig. 2 (NRMSE = 0.078). The error in the estimated laboratory SMC values represent the variation in the model estimated SMC over sensor zenith and azimuth direction.

Having validated the inversion methodology in a set of controlled laboratory experiments, we now turn our attention to the retrieval of the SMC from data collected by a UAS-based hyperspectral imaging (HSI) system with coordinated ground truth measurements, focusing particularly on a more realistic bootstrap analysis of models developed from in-scene calibration points and then tested on sequestered test data also within the hyperspectral scenes.

**Figure 3:**
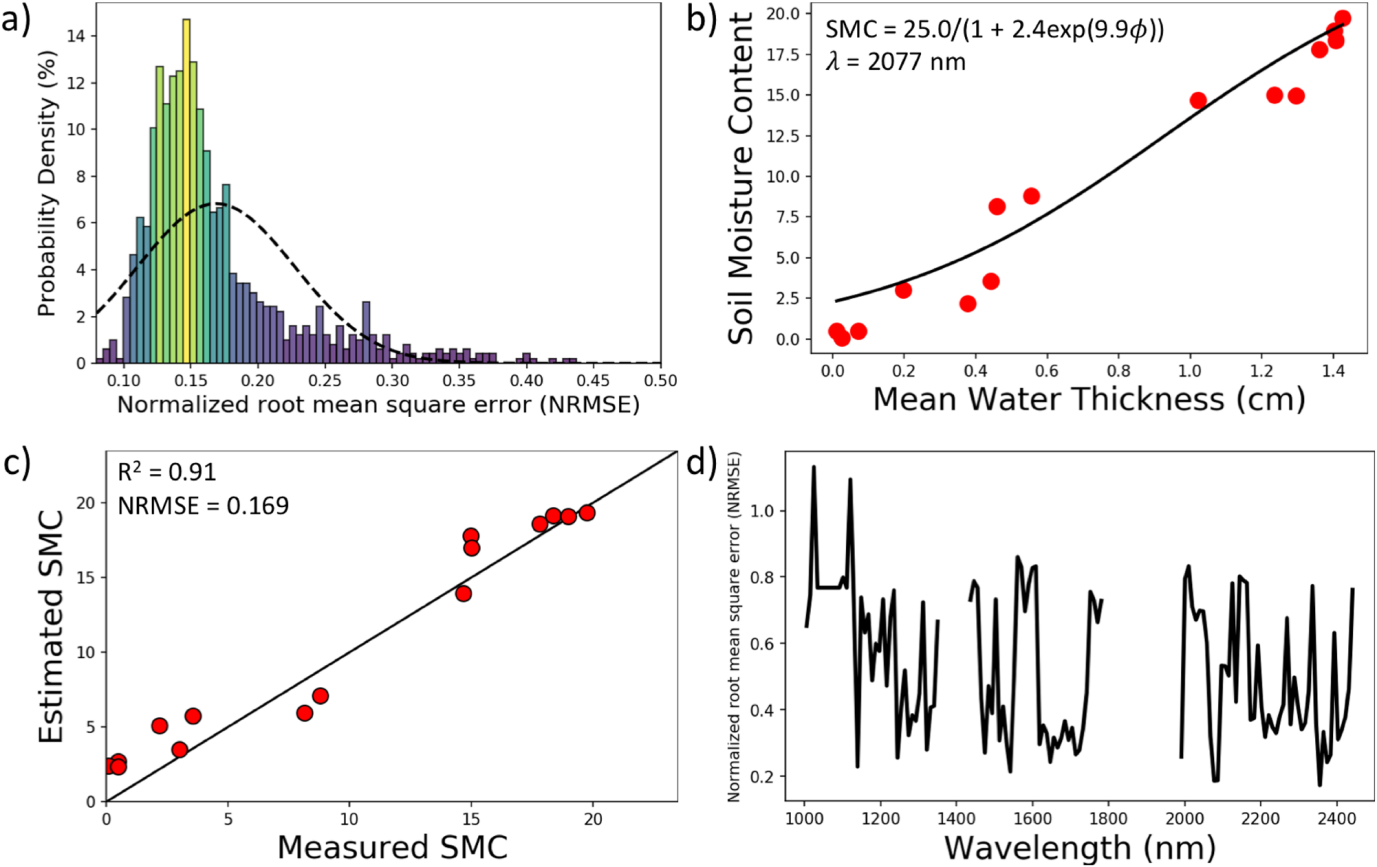
Results of the bootstrap test of the MARMIT model using the SWIR UAS imagery. (**a**) Histogram of the NRMSE value for the relationship between the model predicted SMC of the sequestered test data vs the measured SMC, using 1000 repetitions of the bootstrap test with 50% of the data randomly chosen for calibration and the remaining 50% as the sequestered test data in each trial. Histogram mean $NRMSE = 0.169$ and median $NRMSE = 0.152$ for the test data. (**b**) Calibration of a typical model for $\lambda$ = 2077 nm showing the model (black curve) obtained from the calibration data relating mean water thickness and soil moisture content. Also shown: model outputs for the test data (red points) for this bootstrap trial. (**c**) Typical model output for one of the bootstrap iterations comparing the model estimated SMC for the sequestered test data points against the actual SMC measured in the field. (**d**) Normalized root mean-square error of the selected model shown in (**b**) and (**c**) as a function of wavelength on the test data from that bootstrap iteration, illustrating that multiple wavelengths would provide similar performance.

**Figure 4:**
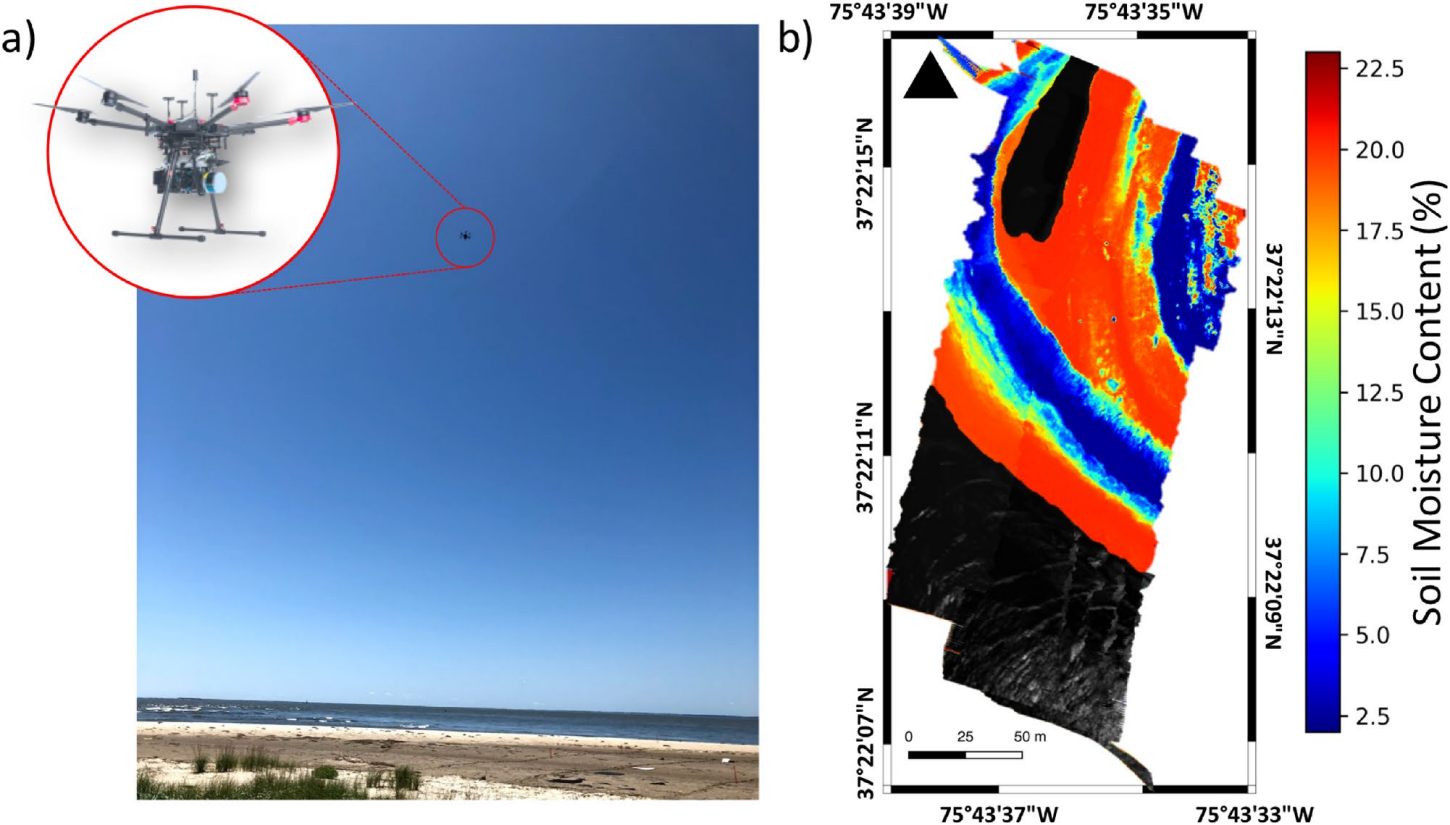
(**b**) MARMIT model fit for a typical model (the one shown in part Fig. 3b applied to a SWIR hyperspectral imagery mosaic collected over the Hog Island beach site on July 14, 2018, showing the retrieved SMC over the entire beach and dune region covered by the drone in one sortie. Wavelength used for the model was 2077 nm. (**a**) Field site, as seen from the dunes, showing beach zones with distinctive contrast in SMC and UAS system overhead. Visible stakes mark some of the ground truth positions used in the study. The wet zone closest to the waterline is not visible in this photo due to the slope of the terrain.The 2018 ground survey points used in this study are located on a 50 m radius around 37$^{\circ }$ 22$^\prime$ 14.45$^{\prime \prime}$ N, 75$^{\circ }$ 43$^\prime$ 36.06$^{\prime \prime}$ W. The survey points from 2019 were $\sim$ 0.25 km from the 2018 site at 37$^{\circ }$ 22$^\prime$ 8.65$^{\prime \prime}$ N, 75$^{\circ }$ 43$^{\prime}$ 28.16$^{\prime \prime}$ W.

We collected hyperspectral data along the shore on the southern tip of Hog island, VA, using UAS-based imaging platforms^15^ during field campaigns in 2018 and 2019^33^. From the UAS platforms, we collected hyperspectral imagery from two HSI systems, one a Headwall Nano-Hyperspec with 270 spectral bands in the visible and near infrared (0.4–1.0 $\upmu {\text {m}}$), and the second a Headwall Micro-Hyperspec with 267 spectral bands in the shortwave infrared (SWIR) (0.9–2.5 $\upmu {\text {m}}$) . However, based on the results of our lab experiments, we decided to use only the SWIR hyperspectral imagery to perform the inversion of the MARMIT model to retrieve SMC. A typical logistic function relating ground measurements of SMC to mean equivalent water thickness of a calibrated MARMIT model appears in Fig. 3b. For the bootstrap trial shown in Fig. 3b, we found that the best agreement between the model output SMC for the calibration data and the ground truth at these sites occurred at a wavelength of 2077 nm with NRMSE value of 0.084, a result similar to the result obtained for the beach data in our lab study (Fig. 1d). To assess the validity of the MARMIT approach to estimating SMC, we undertook a bootstrap analysis, partitioning randomly selected pairs of SMC field measurements and associated UAS SWIR hyperspectral reflectance data into separate calibration and sequestered test sets for evaluation of the model as described in the “Methods” section. The bootstrap trials consisted of calibration and sequestered test data sets of equal size. For each bootstrap trial, we calibrated both the MARMIT model and choose the wavelength using the calibration data set, with the selected wavelength corresponding to the case of best agreement between model output SMC and ground truth SMC over the calibration data set in the trial. Then we evaluated the model independently on the sequestered test data set of the bootstrap trial. A histogram (Fig. 3a) of the sequestered test set results over 1000 bootstrap trials illustrates that the SMC model estimates from the UAS SWIR hyperspectral system agree well with the measured field SMC at the sequestered test sites with mean NRMSE = 0.169 and median NRMSE = 0.152 and with the majority of trials having NRMSE values between 0.1 and 0.25 for the sequestered test data. The heavy tail of the distribution certainly results from the limited number of field samples available for calibration and testing in each trial, a total of 26 positions evenly split between the two sets in each bootstrap iteration. Figure 3b illustrates a typical bootstrap MARMITforSMC calibration curve. Figure 3c uses the same calibrated model illustrated in Fig. 3b and now compares the model estimated SMC for the sequestered test data to the measured field SMC. A retrospective analysis following the test result in Fig. 3c compares what would have happened had a different wavelength been selected at the calibration stage: the NRMSE as a function of wavelength for the test data set appears (Fig. 3d); here we have removed the atmospheric absorption bands. Similar to our observations from the lab, there are several wavelengths in the 1000–2500 nm range with small NRMSE suitable for retrieving SMC with good accuracy. To illustrate performance of the model over the study area, we used the MARMIT model for which the derived logistic function appears in Fig. 3b to map SMC across our beach study site. The resulting SMC superimposed on a mosaic of the UAS hyperspectral imagery appears in Fig. 4. The SMC map was derived using the 2077 nm band, which gave us the best fit for the logistic function on the calibration data of the bootstrap trial in Fig. 3b. Beyond our specific knowledge of the ground validation sites used in calibrating and testing our model, we know from visual inspection during the field surveys that the sediment close to the shoreline had a higher water content, followed by a region of low, high and again low SMC moving away from the shoreline toward the dunes. A ground photo of the study site appearing in Fig. 4 illustrates these sediment moisture zones which the retrieved SMC map, also shown in Fig. 4, delineates.

## Discussion

The change in spectral reflectance with varying moisture content of the soil is generally based on two different processes: (1) the internal reflection of the incident photons between the water and soil layer, resulting in the darkening of the soil, and (2) the absorption of the incoming photon by the water layer^34^. In this study, we use a physics based model, MARMIT^26^, that takes into account these two phenomena in expressing the effect of soil moisture content. Detailed in the “Methods” section, MARMIT is a recent improvement to the work of Bach and Mauser^34^ in modeling reflectance of soils under varying moisture conditions.

A number of soil properties also influence reflectance^35,36^. Among others, these include composition, density, grain size distribution, and roughness. While it is true that MARMIT does not quantify surface roughness explicitly, roughness is implicitly embedded in the reflectance of the soil, for example, in the dry reflectance component of the model. The presence of water obviously may change the roughness of the soil surface, and to some extent the “equivalent” nature of the water thickness layer in MARMIT potentially accounts for this property of the soil. Relationships such as these should be further explored in future studies. While there are some radiative transfer models such as those due to Hapke^36,37^ that explicitly attempt to account for macroscopic roughness, some of our past studies^38^ and those of other authors^39^ have found significant discrepancies, especially in the forward scatter direction^38^. These are areas that require active continued research. In their original article introducing MARMIT, Bablet et al.^26^ suggested that parameters in the SMC sigmoidal calibration curve may be related to other important geophysical parameters that describe soil structure and influence soil reflectance. For example, the asymptote K in Eq. (5) is likely correlated with sediment porosity^26^. Other radiative transfer models such as those due to Hapke explicitly parameterize sediment fill factor^36,40^, and we have successfully used a variant of this model to retrieve sediment fill factor from multi-view hyperspectral imagery^33,41^; however, models such as these have more free parameters that must be optimized.

We have focused on the retrieval of the soil moisture content through the inversion of MARMIT from five different soil data sets. Four of these sediment data sets involved the validation of the MARMIT model under controlled laboratory conditions. The four sets of laboratory samples are from distinctly different soil types with varying physical characteristics such as mineralogy, salinity, texture, organic matter content and roughness. We routinely perform geotechnical analyses with our soil samples acquired in our field campaigns to determine parameters such as grain size distribution, moisture content, and bulk density, among others. In this study, we focus primarily on the soil moisture content. The laboratory experiments examined the dependence of the retrieval of SMC on wavelength and relative sensor azimuth. The SMC estimates from the MARMIT model fit the measured laboratory SMC with high accuracy for all four sediment samples. We developed model fits with MARMIT for the SMC independently for each sediment sample and on a per wavelength basis. While we obtained the best result at a different wavelength for each of the four soil data sets, with the NRMSE value less than 0.145, there were numerous wavelengths in the SWIR where the retrieved SMC was highly accurate across all four data sets. Overall the SWIR model fit represented by the NMRSE was more accurate than in the VIS-NIR portion of the spectrum. Although the shape of the BRDF for sediments varied substantially with the presence of water, the inversion methodology to retrieve SMC was not substantially affected by the sensor relative azimuth (sensor azimuth relative to illumination orientation).

Following our laboratory validation studies, we modeled SMC from data collected by a UAS-based SWIR HSI sensor. Our area of study was the shoreline on the southern tip of Hog island. The MARMIT fit to SMC calibration data had a NRMSE = 0.084 similar to the lab experiments, while independent tests of model performance using a bootstrap analysis achieved on average an agreement between predicted SMC and field measured SMC with NRMSE = 0.169 over 1000 bootstrap trials and a median agreement of NRMSE = 0.152. The MARMIT model provided us a way to map moisture content on the shore of Hog Island with data collected from a UAS-mounted SWIR HSI sensor. While MARMIT does require a calibration step, the fact that spatially and temporally disjoint data taken $\sim$ 0.25 km and a full year apart could be used successfully in the bootstrap validation analysis of this work, suggests that it should be feasible to calibrate models to specific regions. In the future, the well-documented ability of hyperspectral data to be used to distinguish soil types^42^ offers a paradigm for how this might be used in an end-to-end system, where in the first stage of processing, spectral signatures would be used to filter data into appropriate regional MARMIT models. Thus, future studies should focus on extending the retrieval of SMC using MARMIT over a variety of soil types with several different physical properties (composition, roughness, grain size, density, texture, etc.), to investigate the fidelity of MARMIT to characterize SMC over a wider range of possible surface conditions and soil types, and the validity of such region-specific models should be tested with hyperspectral imagery and appropriate ground truth as has been done in this work.

## Methods

### MARMIT model

The MARMIT^26^ belongs to a category of radiative transfer models sometimes referred to as “equivalent slab models”^36^. MARMIT traces its roots historically to the work of Ångström who was the first to model the “darkness” of wet soil in 1925^26,43^. Ångström explained this phenomena using a first order approach, where the diffuse reflection from a rough surface results in total internal reflection at the liquid–air interface of the water layer covering the surface. The total internal reflection increases the absorption of light by the particles, which produces the darkening effect of wet soil^34,43^. Lekner and Dorf (L&D) in 1988^44^ further improved Ångström’s model for wet surfaces by incorporating the effects of spectral reflectance and the influence of the wavelength dependency of the index of refraction of water^26,34,44^. L&D assumed water absorption is negligible in the VIS-NIR. This is a limiting assumption because the model cannot, therefore, be applied in the SWIR, where there are strong absorption bands for water. In 1994^34^, Bach and Mauser introduced absorption features into the reflectance model, using the Beer–Lambert model to describe transmittance within the water layer. The MARMIT model introduced by Bablet et al.^26^ is a recent improvement to the Bach model accounting for the transmittance of light across the liquid–air layer. Their model expresses the total reflectance of the wet soil ($R_{ws}$) as a summation of the successive reflections and refractions at the surface:

1 $$\begin{aligned} R_{ws} = r_{12} + \frac{t_{12}t_{21}R_{d}T^2_{w}}{1 - r_{21}R_{d}T^2_{w}}, \end{aligned}$$

where $r_{12}$ and $t_{12}$ are the unpolarized Fresnel reflection and transmission coefficients at the air–liquid interface respectively, $r_{21}$ and $t_{21}$ are the unpolarized Fresnel reflection coefficient and transmission coefficients at the liquid–air interface respectively, $R_d$ is the reflectance of dry soil, and $T_{w}$ is the transmittance of the light ray calculated using the Beer–Lambert law:

2 $$\begin{aligned} T_w =e^{-\alpha _B \times L}, \end{aligned}$$

where $\alpha _B$ $[m^{-1}]$ is the specific absorption coefficient of water and *L* [*m*] is the thickness of the equivalent water layer. To obtain the unpolarized Fresnel coefficients for $r_{12}$, $t_{12}$, $r_{21}$, and $t_{21}$, the model uses averages of the parallel and perpendicular components of the Fresnel coefficients. The MARMIT model also represents the soil surface reflectance ($R_{mod}$) as a combination of wet and dry areas, by introducing an efficiency parameter ($\varepsilon$), to represent the fraction of wet soil present:

3 $$\begin{aligned} R_{mod} = \varepsilon R_{ws} + (1 - \varepsilon ) R_{d}. \end{aligned}$$

### Inversion methodology

The MARMIT inversion methodology for soil moisture content (MARMITforSMC)^26^ minimizes the residual between the estimated $R_{mod}$ (Eq. 3) and measured total reflectance $R_{measured}$ of the wet soil for acquired hyperspectral reflectance data at different moisture levels. In our study, the minimization also involved hyperspectral data acquisition over different illumination and sensor view geometries, in both laboratory and field settings. The minimization employs a two-parameter search over the thickness of the water level, *L* [*m*], and the efficiency factor, $\varepsilon$, using the gradient based Nelder–Mead simplex method^45^. MARMITforSMC^26^ compares the measured soil moisture content (SMC) with the mean equivalent water thickness, $\phi$, which Bablet et al.^26^ identify as the mean light path, defined by:

4 $$\begin{aligned} \phi = L \times \varepsilon . \end{aligned}$$

MARMITforSMC assumes that a logistic function describes the relationship between SMC and mean water thickness^26^:

5 $$\begin{aligned} SMC = \frac{K}{1 + \alpha e^{ - \psi \phi }}, \end{aligned}$$

where K is the maximum (asymptotic) value of the logistic function, $\psi$ is the steepness of the curve, and $\alpha$ is a translational factor along the x-axis (equivalent water thickness $\phi$). Bablet et al.^26^ suggest, and provide some supporting evidence, that the free parameters of the sigmoid calibration step may be related to geophysical properties of the sediment: for example, *K* may depend on sediment porosity^26^.

The calculation of the reflectance for wet soil using the MARMIT model requires the spectral index of refraction for water, based on values tabulated by Segelstein^46^. We also need the spectral absorption coefficient of water, derived by Pope and Fry^47^ and Kou et al.^48^. These values for the water absorption coefficients and refractive index are available online^49^.

### Experimental design

Our laboratory experiments included bi-conical reflectance factor (BCRF)^50^ measurements of four different sediment samples using GRIT-T^32^. The four samples represent distinctly different soil types, varying in physical characteristics such as mineralogy, salinity, texture, organic matter content and roughness. The samples were acquired from the Algodones Dunes^51,52^ in California, a lakebed region in Northwest Nevada^53^, and Hog Island, VA^33,54^ in the Delmarva Peninsula of Virginia. The Algodones sediment sample (ALG) is typically composed of quartz, feldspar, rock fragment and an assortment of heavy minerals^55^. The Nevada sample (NEV) is mostly composed of clay. One of the sediment samples from Hog Island is from a salt-panne (HOGP) environment^33^ and soil from this region can be considered to be a loam, consisting of mostly clay, silt and sand. Like the ALG desert sediment, the other sample from the island is also a sandy sediment taken from the beach shore on Hog Island (HOGB). In the experiments, we varied the soil moisture content of the samples using a Cornell Sprinkle Infiltrometer^56^ to determine the effect on observed BCRF.

We initially dried each of the four samples over a 24 h period at $110\;^{\circ }$C to remove all moisture from the sediment. To ensure that all samples in our experiments had approximately the same initial dry density, we prepared the samples using a dry pluviation process^57,58^. The drop height of the sediment within the pluviation apparatus is correlated with the relative density of the sediment. Thus, by maintaining the same drop height for all our samples, we could ensure approximately the same initial dry density of the sample.

The four samples then underwent BCRF measurements using GRIT-T. Following the BCRF measurements with the dry samples, we introduced water into the sample using a sprinkle infiltrometer. The sprinkle infiltrometer is a rainfall simulator^59^, which introduces moisture into the sample at a wide range of predetermined rates. The apparatus wets the soil in a more natural manner and removes the possibility of soil slaking due to the introduction of water. The instrument also creates a realistic boundary condition for the soil layer, which includes the effect due to roughness of the soil sample that can greatly influence the infiltration process^59^. The saturated soil sample then underwent BCRF measurements using GRIT-T^32^, and we continued to collect BCRF measurements every few minutes as the sample slowly air-dried, reducing its SMC. For each soil sample, we achieved approximately 20 BCRF measurements for different levels of SMC. This data was the basis for the retrieval of SMC from inversion of the MARMIT model described above. The BCRF measurement from the dry sample served as the dry reflectance $R_d$ described in Eq. (3).

Following the laboratory studies, we applied the MARMIT model to extract soil moisture content from remotely sensed hyperspectral imagery collected from a UAS during field campaigns in 2018 and 2019. We undertook the field campaign at Hog Island (37$^{\circ }$ 25$^\prime$ 5.91$^{\prime \prime}$ N, 75$^{\circ }$ 41$^\prime$ 36.71$^{\prime \prime}$ W), a barrier island off the coast of Delmarva Peninsula, Virginia. The island is part of the Virginia Coast Reserve/Long Term Ecological Research (VCR/LTER) site^60,61^, which has been involved in extensive ecological and geological studies^62–68^. Hog Island bounds a shallow coastal bay, 14 km off the mainland, and is approximately 10 km long and 2.5 km in width.

This study was part of a broader field campaign conducted in summers of 2018 and 2019 to study the beach and intertidal zone sediments^33^ as well as to characterize and map the salt marsh ecosystem^69,70^ in the southern portion of Hog island. The data collected for this study took place specifically along the shore on the southern tip of the island. We imaged the region of interest (ROI) of our study using two different hyperspectral imaging (HSI) sensors onboard a UAS. One of the sensors was a Headwall Nano-Hyperspec, which is a pushbroom system providing spectral measurements from 400 to 1000 nm with 270 spectral bands and 640 across-track spatial pixels. We also imaged using a Headwall Micro-Hyperspec SWIR sensor, another pushbroom system providing spectral measurements from 900 to 2500 nm with 267 spectral bands and 384 across-track spatial pixels. Contemporaneously, we collected extensive ground validation data, including SMC, all georeferenced over our ROI using a Trimble R10 kinematic GPS. The ground truth SMC data collected at positions within the field of view of the hyperspectral imagery served as a validation set for the bootstrap analysis that we reported in the “Results” section. For each iteration of the bootstrap analysis, we randomly partitioned pairs of field SMC data and corresponding UAS hyperspectral SWIR reflectance spectra into two sets: a calibration set and a sequestered test set, with 50% of the paired field positions and spectra assigned to the calibration set (13 points) and the remaining 13 pairs assigned to the sequestered test set. The data from one additional field position, not part of the calibration or test pair sets, served as the dry reflectance $R_d$ in Eq. (3). During the calibration phase of each bootstrap iteration, we used the calibration pairs of UAS reflectance and ground SMC to implement the Nelder–Mead optimization of the free parameters *L* and $\varepsilon$ and to fit the logistic relationship between SMC and equivalent water thickness $\phi$ (Eq. 5). Across wavelength, we selected the best model for evaluation by determining the wavelength for which there was the best agreement between the optimized MARMIT model SMC output for the calibration data set and field SMC. Once we had completed the calibration and wavelength selection using the calibration data set, we then used the sequestered test data pairs to evaluate the model in comparison to the measured ground truth at these test positions in each bootstrap trial. In this study, we used 1000 bootstrap trials to develop the evaluation statistics for the MARMIT model.

## Data Availability

The BCRF dataset of four different sediment samples generated and analysed during the current study are available at https://dx.doi.org/10.35009/cfccis-7c48.
